# Evaluation of the Bio-Rad Geenius HIV 1/2 Confirmation Assay as an Alternative to Western Blot in the Korean Population: A Multi-Center Study

**DOI:** 10.1371/journal.pone.0139169

**Published:** 2015-09-30

**Authors:** Hee-Won Moon, Hee Jin Huh, Gwi Young Oh, Sang Gon Lee, Anna Lee, Yeo-Min Yun, Mina Hur

**Affiliations:** 1 Department of Laboratory Medicine, Konkuk University School of Medicine, Seoul, Korea; 2 Department of Laboratory Medicine, Dongguk University Ilsan Hospital, Goyang, Korea; 3 Eone Laboratories, Incheon, Korea; 4 Green Cross Laboratories, Yongin, Korea; 5 Seoul Clinical Laboratories, Yongin, Korea; The University of Hong Kong, HONG KONG

## Abstract

Recently updated recommendations for diagnosis of HIV infection suggest a new diagnostic algorithm including HIV-1/HIV-2 antibody differentiation immunoassay instead of western blot (WB) as a confirmatory testing. We evaluated Bio-Rad Geenius HIV1/2 confirmation assay as a simple and reliable alternative to WB in the Korean population with low HIV prevalence. The Geenius HIV1/2 was performed in a total of 192 serum specimens (140 reactive and 52 nonreactive specimens by ARCHITECT HIV Ag/Ab Combo assay) that were prospectively collected from five institutions. HIV-1 nucleic acid amplification test (NAT) was performed in negative or indeterminate specimens by Geenius HIV1/2 or WB. Among 140 reactive specimens by HIV Ag/Ab assay, 82 (58.6%) were positive for HIV-1 Ab by Geenius HIV1/2. Among 58 negative or indeterminate specimens by Geenius HIV1/2, four specimens (6.9%) were positive by HIV-1 NAT. The sensitivity and specificity of Geenius HIV1/2 were 95.3% and 100.0%, respectively. When we considered only WB, the sensitivity and specificity of Geenius HIV1/2 were 100.0% and 99.1%, respectively. Agreement between Geenius HIV1/2 and WB was excellent (weighted Kappa = 0.89). The Geenius HIV1/2 is simple and time-saving compared with WB. It has an excellent performance and can be a reliable alternative to WB. HIV-1 NAT should be performed in negative or indeterminate specimens by Geenius HIV1/2 to detect acute HIV infection as recommended in new HIV testing algorithms.

## Introduction

Globally, there were 1·8 million new human immunodeficiency virus (HIV) infections, 29·2 million prevalent HIV cases, and 1·3 million HIV deaths in 2013 [1]. Accurate laboratory diagnosis of HIV infection is important for identifying persons who could benefit from treatment and for reducing HIV transmission. In many countries, traditional algorithm for diagnosis of HIV infection has been a two-step process, including an initial testing with conventional immunoassay or rapid test and a more specific supplemental test such as western blot (WB) and immunofluorescence assays (IFA) [2]. The performance of initial enzyme immunoassay (EIA) has improved significantly, and current fourth-generation EIA combines detection of immunoglobulin (Ig) G and M antibodies with HIV p24 antigen detection (HIV antigen/antibody combination assay, HIV Ag/Ab assay), allowing further reduction of window period[3, 4].

Korea is a country with low HIV prevalence; the annual HIV seroprevalence was 13–15 per 100,000 individuals [5], and the incidence of new HIV infection was 552 in 2013 [1]. The national strategy of HIV testing in Korea has been a two-step process, in which positive results by initial EIA (at public health centers and private medical centers) are confirmed by WB in the National Institute of Health [6, 7].

Recently, updated recommendations for diagnosis of HIV infection by the US Centers for Disease Control and Prevention (CDC) and the Association of Public Health Laboratories (APHL) suggest a new diagnostic algorithm [8]. It includes initial Ag/Ab assay followed by HIV-1/HIV-2 antibody differentiation immunoassay instead of WB for confirmatory testing. This approach with a rapid and simple differentiation immunoassay would decrease reporting time, identify patients with acute HIV infection earlier, and improve patient care [9, 10]. The Bio-Rad Multispot HIV-1/HIV-2 (Bio-Rad Laboratories, Redmond, WA) is a US FDA-approved HIV-1/HIV-2 differentiation assay; however, this kit is available only in the United States. The Bio-Rad Geenius HIV1/2 confirmation assay (Bio-Rad, Marnes-la-Coquette, France, Geenius HIV1/2) also received US FDA approval in October, 2014 and is available worldwide.

## Objective

We evaluated the Geenius HIV1/2 as a rapid, simple, and reliable alternative to WB in the Korean population. To the best of our knowledge, this is the first evaluation of the Geenius HIV1/2 in an Asian population with low HIV prevalence.

## Materials and Methods

### Specimens

A total of 192 serum specimens were collected from two university hospitals (Konkuk university medical center and Dongguk university Ilsan hospital) and three reference laboratories in Korea. All serum specimens were initially determined by the 4th generation HIV Ag/Ab assay (ARCHITECT HIV Ag/Ab Combo assay, Abbott Laboratories, Abbott Park, IL), and when reactive, we performed duplicated tests. All repeatedly reactive specimens by the ARCHITECT HIV Ag/Ab Combo were prospectively collected. We could recruit 140 reactive specimens and these specimens were submitted to WB based on a conventional algorithm (81 WB positive, five WB indeterminate, and 54 WB negative). The 52 nonreactive specimens by ARCHITECT HIV Ag/Ab Combo were additionally selected, including five with high titer autoantibody, five with positive HCV antibody, and seven with positive CMV DNA. Each serum was stored at −70°C until analysis.

All serum specimens were tested by the Geenius HIV1/2. According to the new recommendation, sera with reactive HIV Ag/Ab assay but nonreactive or indeterminate Geenius HIV1/2 results were further tested with HIV-1 nucleic acid amplification test (NAT). Sera with discrepant results between WB and Geenius HIV 1/2 were also tested with HIV-1 NAT. The diagnostic performances of Geenius HIV1/2 were evaluated based on the results of initial HIV Ag/Ab assay, WB, and HIV-1 NAT. The 52 nonreactive specimens by HIV Ag/Ab assay and 54 negative specimens by WB and HIV-1 NAT were considered negative for HIV infection. The 81 WB-positive and five HIV-1 NAT-positive specimens were considered positive for HIV-1 infection. The study protocol was approved by the Institutional Review Board of Konkuk University Medical Center, Seoul, Korea.

### HIV assays

The ARCHITECT HIV Ag/Ab Combo is a chemiluminescent magnetic microparticle-based immunoassay used to determine the presence of HIV-1 p24 antigen and antibodies to HIV-1 group M, group O, and HIV-2. Specimens with signal to cut-off (S/CO) values greater than or equal to 1.0 were considered reactive, and specimens with S/CO values less than 1.0 were considered nonreactive. WB was performed using MP Diagnostics [MPD] HIV BLOT 2.2 (MP Biomedicals Asia Pacific Pte Ltd., Singapore Science Park, Singapore), which detect HIV-1 and HIV-2 antibodies. Interpretation was performed according to the manufacturer’s recommendation, and detection of two ENV HIV-1 positive (gp160/gp41 and gp120) and GAG (p17, p24, p55) or POL (p31, p51, p66) was considered HIV-1 positive.

The Geenius HIV-1/2 assay is a recently-launched, single-use immunochromatographic test. It is intended for confirmation and differentiation of HIV-1 and HIV-2 in the positive specimens by screening test. This kit utilizes immobilized HIV-1 (p31, gp160, p24, and gp41) and HIV-2 (gp36 and gp140) antigens for the detection of antibodies to HIV-1 and HIV-2 in serum, plasma, or whole blood. The band patterns were read manually by two experts, and the interpretation criteria were any two bands of four test lines with at least one envelop peptide (gp160 or gp41) for HIV-1 positive and two HIV-2 bands for HIV-2 positive. Others were considered indeterminate, and a test without distinct control band was regarded as an invalid test.

HIV-1 NAT was performed using *artus* HI Virus-1 QS-RGQ Kit (QIAGEN, Hilden, Germany), which detects HIV-1 RNA using real-time polymerase chain reaction (PCR). We used QIAsymphony (QIAGEN) for specimen preparation, and PCR was performed using Rotor-Gene Q Instrument (QIAGEN).

### Statistical analysis

The sensitivity and specificity of each assay (with 95% confidence interval [CI]) were determined. Agreement between the Geenius HIV1/2 and WB was assessed using Cohen’s Kappa (agreement: < 0.4, poor; 0.4–0.75, fair to good; > 0.75, excellent) [11]. Statistical analysis was performed using MedCalc Statistical Software (version 12.3.0, MedCalc Software, Mariakerke, Belgium).

### Results

**Fig 1:**
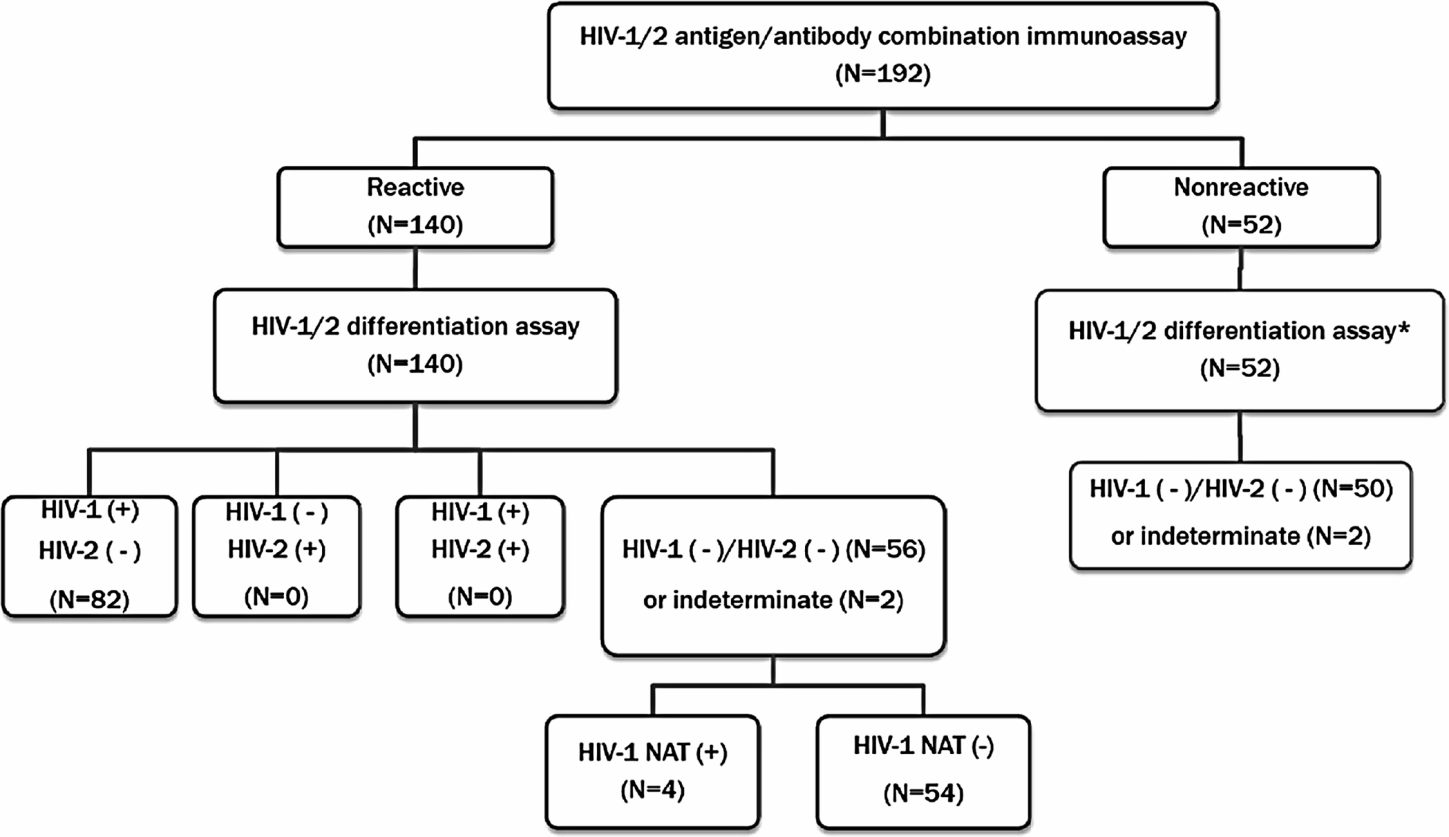
Distribution of results based on new HIV diagnostic algorithm. *The 52 specimens with nonreactive HIV Ag/Ab assay were additionally tested with HIV-1/HIV-2 differentiation assay, although no further testing is required in the new recommendation.

The results of this study based on new recommendations are presented in **Fig 1**. According to the new recommendations, 140 reactive specimens by HIV Ag/Ab assay were tested by HIV-1/HIV-2 differentiation assay (Geenius HIV-1/2), and 82 (58.6%) were positive for HIV-1 Ab. In the 58 (41.4%) specimens with negative or indeterminate results by HIV-1/HIV-2 differentiation assay, four specimens showed positive results by HIV-1 NAT. The 52 specimens with nonreactive HIV Ag/Ab assay were additionally tested with HIV-1/HIV-2 differentiation assay, although no further testing is required for those specimens in new recommendations. Among them, two specimens showed one faint band, and the others showed nonreactive results by HIV-1/HIV-2 differentiation assay.

Overall performances of the Geenius HIV1/2 are presented in **Table 1**. When confirmation was based on both WB and HIV-1 NAT, the sensitivity and specificity of the Geenius HIV1/2 were 95.3% and 100.0%, respectively. There were four false-negative specimens, which were nonreactive or indeterminate by Geenius HIV1/2 and positive by HIV-1 NAT. When we considered only WB, the sensitivity and specificity of the Geenius HIV1/2 were 100.0% and 99.1%, respectively. One specimen was reactive with Geenius HIV1/2 and indeterminate with WB. Since we prospectively collected reactive specimens by HIV Ag/Ab assay due to the low HIV prevalence, predictive values were not calculated.

**Table 1:** Overall performances of Geenius HIV1/2 assay based on the results of WB and HIV-1 NAT*.

Confirmation method	Sensitivity (95% CI)	Specificity (95% CI)	TP	FP	TN	FN	Total
WB and HIV-1 NAT	95.3% (88.5–98.7)	100.0% (96.5–100.0)	82	0	106	4	192
WB	100.0% (95.5–100.0)	99.1% (95.1–99.8)	81	1	110	0	192

*Indeterminate results were considered negative.

Abbreviations: WB, western blot; NAT, nucleic acid amplification test; CI, confidence interval; TP, true positive; FP, false positive; TN, true negative; FN, false negative.

The results between WB and Geenius HIV1/2 are compared in **Table 2**. Agreement between the two assays was excellent (weighted Kappa = 0.89). Seven specimens showed discrepant results between WB and Geenius HIV1/2 (**Table 3**). There were two nonspecific reactions in Geenius HIV1/2; four acute HIV infections; and one indeterminate result of WB in HIV infection.

**Table 2:** Comparison of the results between WB and Geenius HIV1/2 assay.

	Geenius HIV1/2			
WB	Positive	Indeterminate	Negative	Total
Positive	81	0	0	81
Indeterminate	1	0	4	5
Negative	0	2	52	54
Total	82	2	56	140
Weighted Kappa (95% CI)	0.89 (0.82–0.97)			

Abbreviations: WB, western blot; CI, confidence interval.

**Table 3:** Specimens with discrepant results between WB and Geenius HIV1/2 assay.

No.	WB	Geenius HIV1/2	Architect S/CO	HIV-1 NAT	Interpretation
1	Negative	Indeterminate (very weak band)	1.79	Negative	Nonspecific band of Geenius HIV1/2
2	Negative	Indeterminate (very weak band)	5.25	Negative	Nonspecific band of Geenius HIV1/2
3	Indeterminate	Negative	280.0	Positive	Acute HIV infection
4	Indeterminate	Negative	11.18	Positive	Acute HIV infection
5	Indeterminate	Negative	7.13	Positive	Acute HIV infection
6	Indeterminate	Negative	8.73	Positive	Acute HIV infection
7	Indeterminate	Positive	54.58	Positive	HIV infection

Abbreviations: WB, western blot; S/CO, signal to cut-off value; NAT, nucleic acid amplification test.

## Discussion

For several decades, WB has been the gold standard as a confirmation assay after reactive EIA for HIV antibody in many countries. However, WB is labor-intensive, time-consuming, and requires training and expertise in its interpretation [8]. WB also produces indeterminate results frequently and causes delayed reporting.

The newly recommended algorithm suggests HIV-1/HIV-2 differentiation assay after reactive HIV Ag/Ab assay. There are, however, few commercially available HIV-1/HIV-2 differentiation assays. Although the Geenius HIV1/2 was approved in Europe and the United States, it is not yet approved for *in vitro* diagnosis in some countries. In this study, we wanted to explore the usefulness and reliability of the Geenius HIV1/2 in a population with low HIV prevalence. We performed HIV-1 NAT in all negative or indeterminate specimens by Geenius HIV1/2, according to the new algorithm.

Since we prospectively collected reactive specimens by HIV Ag/Ab assay, we could assess the real distribution of results based on newly recommended algorithm (**Fig 1**). Among the reactive specimens by HIV Ag/Ab assay, 58.6% were HIV-1 reactive by Geenius HIV-1/2 assay and were considered HIV-1 positive.

Overall sensitivity and specificity of the Geenius HIV1/2 based on WB and HIV-1 NAT were 95.3% and 100.0%, respectively, and there were four false-negative results by the Geenius HIV1/2 based on HIV-1 NAT (**Table 1 and Table 2**). Recently, several studies also evaluated the Geenius HIV1/2 and concluded that this kit has excellent performances and many advantages [12–15]. Reported sensitivities and specificities were 99.3–100% and 96.3–100%, respectively [12, 13, 15]. The sensitivity could be lower in studies including specimens of seroconversion or acute infection [12, 16].

Since we evaluated the Geenius HIV1/2 as an alternative to WB and HIV-1 NAT is included as a next step in negative or indeterminate differentiation assay in new algorithms, we could consider WB as a gold standard. Compared with WB, the sensitivity and specificity of the Geenius HIV1/2 were 100.0% and 99.1%, respectively; it means that the Geenius HIV1/2 does not miss WB-positive specimens. Agreement between the Geenius HIV1/2 and WB was excellent (weighted Kappa = 0.89) and could be perfect if we exclude indeterminate results (**Table 2**).

Regarding discrepant results between WB and Geenius HIV1/2, two specimens with negative WB and indeterminate Geenius HIV1/2 were negative for HIV-1 NAT (**Table 3**). One very faint band of Geenius HIV1/2 was observed in these specimens and considered nonspecific positivity. It was considered indeterminate, although the band intensity was very weak, according to the manufacturer’s recommendation. This pattern of faint band in the Geenius HIV1/2 was also observed in nonreactive specimens by HIV Ag/Ab assay (2/52 specimens).

There were four specimens of acute HIV infection in our study population, which constitutes 2.8% among 140 reactive specimens with HIV Ag/Ab assay and 6.8% among 58 specimens with nonreactive or indeterminate with the Geenius HIV1/2 (**Table 3**). In these patients, follow-up tests showed positive conversion of antibody. The main benefit of the new recommendations is to expedite detection of acute HIV cases, and we could find five cases of HIV infection, which showed indeterminate WB results with this algorithm. Importantly, performing HIV-1 NAT should not be omitted when we use new recommendations. However, performing HIV-1 NAT in each clinical laboratory might be difficult and costly than previous algorithms. Since more than 40% of all positive samples require PCR for resolution in our setting with new recommendation, assessment of cost-effectiveness of the modified algorithm is needed in the Asian setting with low HIV prevalence. It is also assessed according to local epidemiology and national policy. European guideline on HIV testing suggests HIV-1 NAT or p24 antigen for unconfirmed specimens [17]. One specimen showed indeterminate WB, positive Geenius HIV1/2, and positive HIV-1 NAT. This could be considered HIV infection and follow-up of WB is needed.

This study is limited in that we could not include HIV-2 positive specimens and could not evaluate the Geenius HIV1/2 for differentiation of HIV-1 and HIV-2. Since HIV-2 infection is endemic in Africa but very rare in Korea, evaluation of the Geenius HIV1/2 without HIV-2 positive specimens could be acceptable for adopting the new diagnostic algorithm in the area of low HIV prevalence. The ability of the Geenius HIV1/2 to differentiate HIV-1 and HIV-2 was reported to be very high [13, 15].

In conclusion, the Geenius HIV1/2 is simple and time-saving compared with WB. It has an excellent performance and can be a reliable alternative to WB. HIV-1 NAT should be performed in negative or indeterminate specimens by the Geenius HIV1/2 to detect acute HIV infection as recommended in new HIV testing algorithms.
